# One-Abutment One-Time Effect on Peri-Implant Marginal Bone: A Prospective, Controlled, Randomized, Double-Blind Study

**DOI:** 10.3390/ma14154179

**Published:** 2021-07-27

**Authors:** Filipe Moreira, Salomão Rocha, Francisco Caramelo, João P. Tondela

**Affiliations:** 1 Institute of Oral Implantology and Prosthodontics, Faculty of Medicine, University of Coimbra, 3000-075 Coimbra, Portugal; 2 Institute of Oral Implantology and Prosthodontics, Faculty of Medicine, University of Coimbra and Center of Research and Innovation in Dental Sciences of the Faculty of Medicine of the University of Coimbra, 3000-075 Coimbra, Portugal; salomaorocha@gmail.com; 3 Laboratory of Biostatistics and Medical Informatics (LBIM), Coimbra Institute for Clinical and Biomedical Research (iCBR), Faculty of Medicine, University of Coimbra, 3000-548 Coimbra, Portugal; fcaramelo@fmed.uc.pt; 4 Faculty of Medicine, University of Coimbra and Center of Research and Innovation in Dental Sciences of the Faculty of Medicine of the University of Coimbra, 3000-075 Coimbra, Portugal; jtondela@fmed.uc.pt

**Keywords:** one abutment one time, implant–abutment connection, marginal bone loss, repeated abutment disconnection

## Abstract

Objective: To evaluate the peri-implant hard tissue change at 6 and 12 months after implant placement between definitive abutment placed at the same time of implant surgery, never removing it, and healing abutment disconnected and reconnected three times until the placement of the final rehabilitation. Material and methods: Each partial edentulous patient could receive between 1 and 4 platform-switched implants in the posterior regions. If the implants had primary stability—implant stability quotient (ISQ) equal to or greater than 50, they were randomized to the test group with the abutment inserted at the same time of implant placement (DA) or to the control group, receiving a healing abutment (PA). At 6 and 12 months after surgery, data related with vertical bone level changes (primary outcome) and other clinical parameters (implant mobility, bleeding on probing, probing depth, plaque index) were assessed. Results: 53 implants were included in the trial and completed 12 months follow-up (overall survival rate: 100%). All implants achieved primary stability, with an average ISQ value of 80.9 on the day of surgery. From surgery to 6 months, the mean bone loss was 0.14 ± 0.18 mm for the DA group and 0.23 ± 0.29 mm for the PA group, without statistical significance difference. Between 6 and 12 months, the mean bone loss was 0.14 ± 0.21 mm for the DA group and 0.21 ± 0.27 mm for the PA group, also without statistical significance between the two groups. There were no statistically significant differences (*p* = 0.330) in total bone loss after 12 months between the control and the study groups. Conclusions: The one abutment one time protocol has at least an equivalent effect on the peri-implant bone level changes when compared with the use of healing abutments that are disconnected and reconnected at least three times.

## 1. Introduction

From the beginning, dental implantology always pursued osseointegration. Successful osseointegration from the clinical standpoint is a measure of implant stability, which occurs after implant integration [1]. One of the most important factors to achieve osseointegration is primary implant stability that can be defined as the mechanical stability between the implant and the surrounding bone [2]. The implant stability quotients (ISQ values) are used as an indicator for mechanical implant stability and are believed to have predictive power for clinical outcome [3].

One of the main goals of modern implantology is the long-term stability of the peri-implant hard and soft tissues in way to achieve and maintain esthetically optimal results [4]. Even if marginal bone loss (MBL) is minimal, it remains clinically relevant mainly because it may be accompanied by soft tissue recession, compromising long-term esthetic, especially if we are considering the anterior region [5].

As a result, many strategies to preserve peri-implant marginal bone level, such as different implant micro and macro designs [6,7,8,9,10,11], surgical and prosthodontic protocols [12,13,14,15], prosthetic superstructures, implant–abutment connections and platform-switching concepts have been developed, although without clear evidence of the superiority of one concept over another [16,17,18], which makes it impossible to state whether the physiological bone remodeling is prosthesis-related, host-related, implant-related or load-dependent, [19] remaining the utilized criteria for success unchanged.

Several authors advocate that the use of a small-diameter abutment compared with the platform diameter of the implant creates an horizontal mismatch between both components, placing the implant–abutment connection away from the bone crest, promoting its maintenance by keeping away the connective tissue inflammatory infiltrate from the peri-implant marginal bone and thus reducing the loading stress in the crestal portion of the bone [20,21,22,23,24]. The concept of platform switching (PS) is still involved in discussion, but it determines changes in clinical practice and implant designs and gave rise to a series of investigations searching for aspects that may influence peri-implant bone level. Factors such as depth of implant placement [18,25,26], type and stability of implant–abutment connections [18,25,26], use of intermediate abutments [27], abutments height [28], soft tissue thickness [29,30], frequent probing during the healing period [31], and repeated disconnections and reconnections of abutments [26,31,32,33] have been associated with variations in peri-implant marginal bone loss.

Besides the implant–abutment interphase, the manipulation of these restorative components may also influence the stability of the surrounding tissues. In the classic clinical protocol for implant-supported rehabilitation, the healing abutment/provisional crown is connected to the implant body, requiring that the latter is exposed to the oral cavity. Before the installation of the final prosthetic restauration, the provisional abutment must be connected and disconnected multiple times for impression taking, substructure try-in, and conformation of the gingival profile. Repeated disconnection and reconnection of the prosthetic components may mechanically compromise the soft tissue barrier, promoting the introduction of bacteria and other contaminants into the implant–mucosa barrier, inducing inflammation, and resulting in an apical shift of the connective tissue attachment and the underlying bone [34].

This experimental evidence prompted the development of the one-abutment one-time protocol, involving the placement of the definitive restorative abutment at the time of implant surgery, thus avoiding its removal during healing. This protocol has been tested in animal models [26,33,35] and in humans mainly in combination with immediate single implant placement [31,36,37]. The scientific evidence on its efficacy when applied to implants placed in healed sites is, however, unclear [38,39].

The objective of our study was to assess the hypothesis that installing the definitive abutment during implant placement at bone level following the PS concept and never removing it would have at least the same impact on the stability of hard and soft peri-implant tissues, when compared with the placing of a healing abutment and subsequent insertion of the definitive abutment only during the restorative phase.

## 2. Materials and Methods

### 2.1. Study Design

The study was designed as a prospective, double blind, randomized, controlled clinical trial (2 centers). The participants were allocated to two groups: test group (immediate definitive abutment - DA), in which the definitive abutments were placed at the same time as the implant surgery and never removed; control group (healing abutment group - PA), in which the abutments were disconnected and reconnected three times throughout the study.

In both groups at the time of the connection of the definitive abutment 3 height measures were used: 1, 2 or 3 mm depending on the evaluation of the mucosa height performed by the surgeon.

Approval by the Ethics Committee of the University of Coimbra was obtained in June 2018 (Nº: CE-048/2018). All participants signed a term of free and informed consent, according to the recommendations of the Declaration of Helsinki.

### 2.2. Study Population

Eligible patients had to fulfill the general study criteria:

Inclusion criteria:

- Minimum of 18 years old;
- At least one adjacent missing tooth in the posterior maxilla or mandible (World Dental Federation positions 4–7) with no free-end unitary implant situation being allowed.
- Patients willing an implant-supported rehabilitation and compromised to attend the follow-up visits.
- Healing post-extraction period of more than 4 months.
- Healed osseous architecture sufficient to receive a VEGA implant^®^ (Klockner Implant System, SOADCO, S.L., Escaldes-Engordany, Andorra) with a diameter and lenght at least of 4 mm and 8 mm, respectively, allowing the existence of a minimum of 1.5 mm bone tissue width surrounding the implant.
- Natural teeth or fixed prosthesis as opposing dentition.
- No signs of periodontal disease: absence of suppuration, plaque index and bleeding less than 15% according to Mombelli’s criteria.

Systemic exclusion criteria:

- Any illness afecting the immunitary system and/or bone metabolism.
- History of administration of bisphosphonates.
- Alterations in coagulation.
- History of radiotherapy of the head or neck.
- Uncontrolled systemic disorders.
- Alcohol and drug abuse.
- HIV+.
- Severe bruxists or with signs of temporomandibular joint (TMJ) pathology or muscle pain.
- Smokers of more than 10 cigarettes per day.
- Factors that, in the investigator’s opinion, complicate the patient’s participation in the study or in data analysis.

Local exclusion criteria:

- Any local pathology that represents a contraindication for the treatment with dental implants.
- Nonhealed alveolar bone.
- Locations submitted to bone regeneration less than 6 months ago
- Bone defect around the contour of the implant requiring regeneration–bone augmentation representing 25% or more of the implant surface in fenestration type defects at the time of implant placement.
- Inadequate oral hygiene
- Active, recurrent or unresolved infections in the area adjacent to the implant installation

Local exclusion criteria after implant placement:

- Lack of primary stability of the implant.
- Inadequate implant position that compromises prosthetic requirements for the study.
- Need for additional regenerative techniques.

### 2.3. Sample Size

The sample size was calculated based on the 0.30 mm clinical margin and assuming a standard deviation in the groups regarding implant placement techniques equal to 0.30 mm, which results in an effect size equal to 1. The level of significance considered was 0.05 and the statistical power 0.90. The sample size was obtained using the proposed expression by Lakens D. [40], having obtained the value of 22 subjects per group.

### 2.4. Implants and Abutments

**Figure 1:**
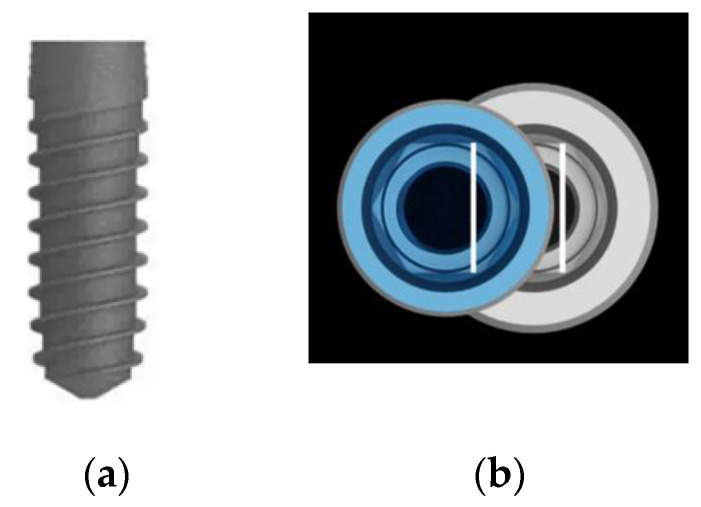
(**a**) Klockner VEGA Surface ContacTi^®^ implant; (**b**) 4.0 and 4.5 mm diameter platform with an internal hexagon connection change of 0.35 and 0.60 mm, respectively.

The bone level implants VEGA Surface ContacTi^®^ (Klockner Implant System, SOADCO S.L., Escaldes-Engordany, Andorra) present conical design, 4.0 or 4.5 mm diameter platform with and an internal hexagon connection change of 0.35 and 0.60 mm, respectively, and 8, 10 and 12 mm in length. The ContacTi surface is a bioactive subtraction surface with thermochemical treatment and a medium roughness Sa 1.6 μ [41]—see Figure 1.

**Figure 2:**
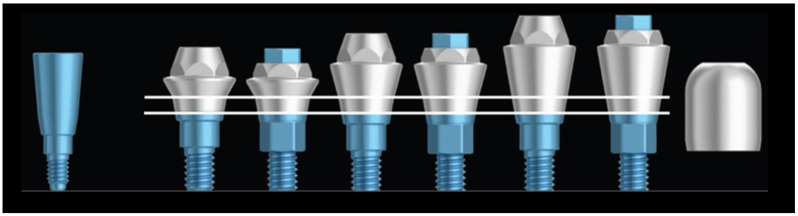
From left to right: healing abutment 5 mm height, Permanent^®^ abutments of 1, 2, and 3 mm height to unitary and multiple rehabilitations, and protective cover for Permanent^®^ abutment.

The healing abutments used had 5 mm high and conical shape, respecting platform-switching concept. The definitive abutments used were Permanent^®^ abutments (Klockner Implant System, SOADCO, Escaldes-Engordany, Andorra) for single or multiple screw-retained restorations, and three measures were available: 1, 2 or 3 mm high, depending on the judgment of the surgeon (Figure 2).

### 2.5. Surgical Procedure and Randomization

One day before surgery, prophylactic antibiotic therapy (amoxicillin or clarithromycin), anti-inflammatory, and gastric protector were prescribed. Surgeries were performed by 2 experienced surgeons (João Paulo Tondela & Salomão Rocha). A crestal incision was made in the keratinized mucosa whenever possible. Full-thickness flaps were raised to expose the bone. Flapless technique was not valid. The surgeon could select the situations in which due to poor bone quality decided to try to increase the primary stability of the implant performing a sub-preparation technique. Sub-preparation was defined as not using the dense bone drill as well as the final pilot drill. Implant shoulder was placed at bone level or subcrestal, and a distance of ≥1.5 mm from the adjacent natural tooth and ≥3 mm between implants was always respected. Crest remodeling was never performed in a way to avoid disruption of the bone ridge. Bone quality found was registered according to the classification of Lekholm and Zarb. The distance from the implant neck to the bone crest was also recorded both mesial and distal with the aid of a manual periodontal probe (PCP UNC15). The implant stability values were recorded with the PenguinRFA™ system (Integration Diagnostics, Göteborg, Sweden). If implants had primary stability, meaning an ISQ ≥ 54 [42,43], they would continue in the study. Healing abuments were connected to all implants before the postoperative radiograph was performed, ensuring the blinding of the examiner responsible for the analysis of the radiographic measurements. After that each implant was allocated randomly to the control group (PA) or test group (DA) by opening sealed envelopes sent directly with each implant. Implants randomized to test group received the definitive Permanent^®^ abutment 1, 2, or 3 mm high, which was fitted with 10 Ncm torque, protected with a titanium cover during the healing period. Implants in the control group remained connected to the conventional healing abutment also with 10 Ncm torque. Implant sites were sutured with non-resorbable sutures obtaining transmucosal healing. Postoperative instructions were given, the patients were instructed not to brush the area until suture removal and to apply 0.12% chlorhexidine gel three times a day. Anti-inflammatory drugs were also prescribed in the postoperatorium (Ibuprofen 600 mg every 12 h).

### 2.6. Prosthetic Procedures

After a healing period of 7–8 weeks, impressions were taken. Screw-retained impression copings closed tray for the Klockner VEGA system^®^ were used. For those implants randomized to control group, healing abutments were disconnected for the first time and impressions were taken at implant level. In the DA group, impressions were taken directly at Permanent^®^ abutment level. In the PA group, the selection of the height of the definitive abutment was performed with the help of the master cast. Within one to four weeks, the metallic substructure was tested. In the PA group, the healing abutments were disconnected for the second time and the try-in was carried out with the definitive abutment that was inserted at that time for this purpose, having returned to the healing abutment after that. Finally, at 12 weeks the healing abutments on the PA were removed for the third and last time. The definitive abutment was installed and torqued to 25 Ncm. The same torque to the Permanent^®^ abutment was established in the DA group (in this group, the definitive abutment was never removed). Both groups received the final screw-retained restorations applying 15 Ncm of torque to the screws.

### 2.7. Clinical Examinations

Clinical examinations were recorded at 6 and 12 months post-surgery follow-up appointments. The parameters modified plaque index (Mombelli) and modified bleeding index [44] were recorded at four sites per implant (mesial, distal, vestibular, and lingual or palatal surfaces) with a periodontal probe (PCP UNC-15). Implant and/or restorations mobility was registered and implant stability was recorded with the PenguinRFA™ system (Integration Diagnostics, Göteborg, Sweden). The ISQ value was measured always to the permanent^®^ abutment when present and directly to the implant in the PA group before the definitive abutment was connected.

### 2.8. Radiographic Assessment

**Figure 3:**
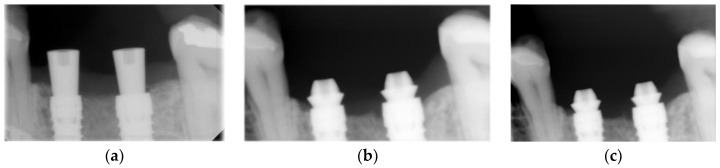
Case evolution control group. (**a**) Radiograph baseline; (**b**) Radiograph 6 months; (**c**) Radiograph 12 months.

**Figure 4:**
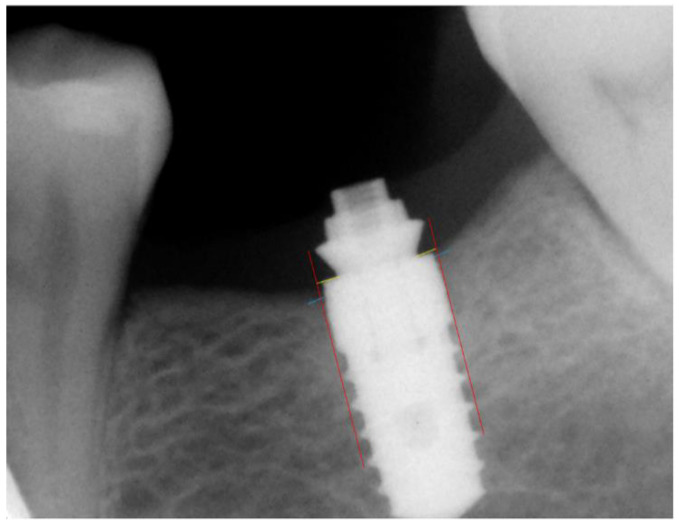
The distance between the implant shoulder and the first bone to implant contact in mesial and distal was used to measure bone level variation throughout time. In yellow: implant platform. In blue: first visible bone contact.

Radiographic examinations were performed before surgery, immediately after the implant placement and at the 6 and 12 month follow-up visits (Figure 3) to evaluate changes in interproximal bone levels using standardized periapical digital radiographs (VisualiXTM GX-S HDI, Gendex Dental Systems, Des Plaines, IL, USA) obtained with a customized sensor holder [45]. Radiographic images obtained were generated on a computer using the VixWinTM program (Gendex Dental Systems, Des Plaines, IL, USA) and stored in compressed JPEG format (Joint Photografic Experts Group) with the resolution of 1368 × 912 pixels. The distance between the implant platform and the first visible bone contact (DIB) was measured for the mesial and distal faces of each implant and the average of these two measurements, rounded to the nearest decimal place, corresponded to the DIB for that implant (Figure 4).

All radiographs were calibrated to adjust their possible distortion by using the known distances between two implant threads and the length of the implant used. Two operators, previously calibrated, performed all the measurements using the ImageJ software package (ImageJ, National Institutes of Health, Madison, MD, USA). Intra-class correlation coefficient determined very good reliability of the measurements obtained by the examiners (ICC = 0.947).

### 2.9. Statistical Analysis

The implant was considered the statistical unit. The equivalence between the two techniques was assessed by the TOST method (Two One-Sided Tests) and by through confidence intervals. Total bone loss was used as the main measure and was determined by the mean of the bone loss in mesial and distal. The same statistical procedure was performed for the bone loss in mesial and distal.

The sample description was performed according to the type of the assessed variable. For qualitative, nominal, and ordinal variables, absolute frequency and relative frequency were used, whereas the quantitative variables were described with means, medians, standard deviation, and percentiles (P25 and P75).

To assess differences in nominal variables between the control and study groups, the Fisher’s exact test was applied.

The comparison over time was performed using the Friedman test after the Shapiro–Wilk test was violated in the assumptions of normality, followed by Wilcoxon post-hoc tests with Bonferroni correction for multiple comparisons.

The comparison between groups of quantitative variables, at each moment, was performed by the Mann–Whitney test when a violation of the assumption of normality was observed and by the t-student test in the opposite case.

The statistical analysis was carried out in R (R Core Team 2017, Vienna, Austria) and in IBM^®^ SPPS^®^ (IBM Corp. Released 2017. IBM SPSS Statistics for Windows, Version 25.0. Armonk, NY: IBM Corp., USA) having adopted a significance level of 0.05.

The null hypothesis of the study considered that there would be no difference in bone loss (or gain) between the 2 groups after implant placement.

## 3. Results

### 3.1. General Information

**Figure 5:**
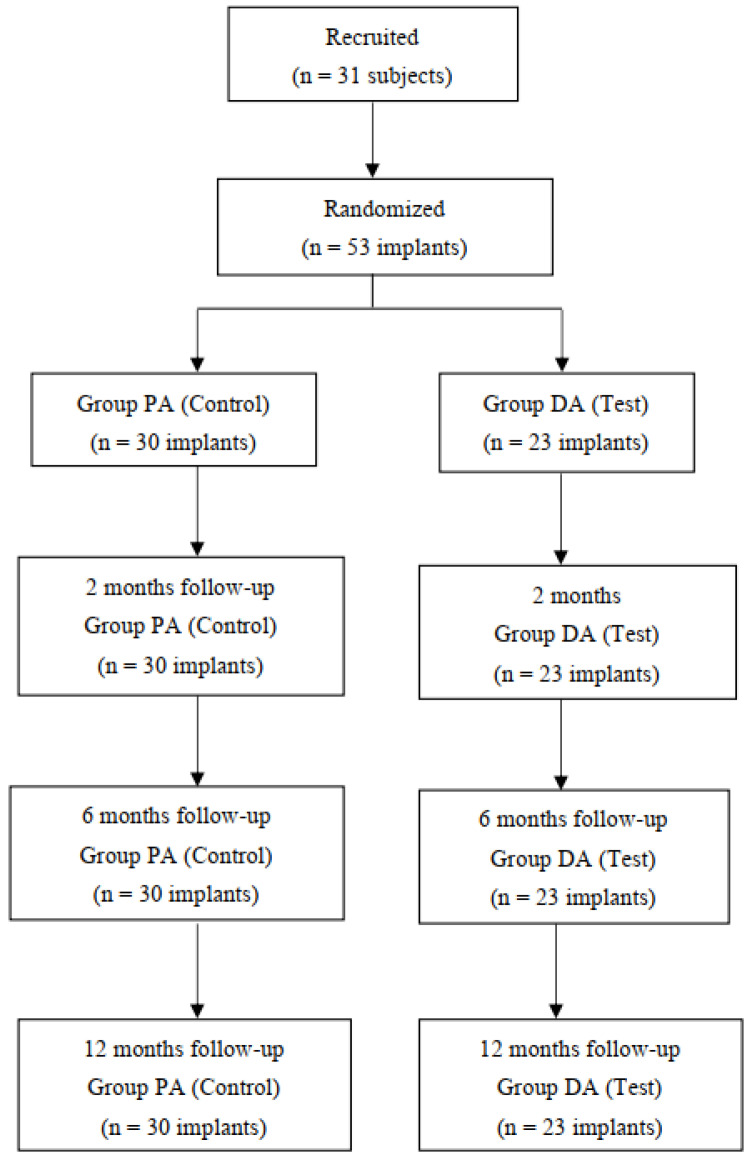
Study sample flow-chart.

At centers 07 and 08 located at the Faculty of Medicine of the University of Coimbra, between January and March of 2017, 31 patients were included, 10 men and 21 women, and 53 implants were installed. No implant was excluded due to lack of primary stability or the need for bone augmentation or impracticability of prosthetic rehabilitation of the implants (Figure 5). All implants underwent the follow-up control at 1–2 weeks, 8–12 weeks, 6 months, and 12 months after surgery, completing the study. Baseline demographic characteristics of the study sample are described in Table 1.

### 3.2. Analysis of Resonance Frequency Values in Both Groups

Primary stability was measured with PenguinRFA™ (Integration Diagnostics Sweden AB, Göteborg, Sweden) and the values recorded in ISQ units (Implant Stability Quotient), ranging from 1 to 100, as observed in Table 2. For the implant to be randomized in one of the groups it should have primary stability, that is, an ISQ-1 greater than 54 [42,43]. Implant stability was also measured on the day of the final impression (ISQ-2), 8 weeks after surgery, and on the 6 (ISQ-3) and 12 months follow-up appointments (ISQ-4). The values’ distribution was very similar between the DA and PA groups.

### 3.3. Changes in the Clinical Outcome Measurements

#### 3.3.1. Modified Plaque Index

The plaque index was assessed on the mesial, buccal, distal, and lingual/palatine surfaces of each crown/abutment. The values obtained for each of the faces were added and divided by 4, in order to obtain the plaque index per implant. At 6 and 12 months follow-up, there were no statistically significant differences between groups (Table 3).

#### 3.3.2. Modified Sulcus Bleeding Index

At 6 and 12 months follow-up, there were no significant differences between the groups related with gingival inflammation (Table 4).

#### 3.3.3. Probing Depth

The probing depth was measured on the mesial, buccal, distal, and lingual/palatine faces of each implant and recorded in mm. The values obtained in each of the faces were added and divided by 4, in order to obtain the probing depth by implant. The probing depth was always lower than 3 mm for both groups and no significant differences between them were observed over time (Table 5).

### 3.4. Total Bone Loss over Time

The radiographic measurement of the mean bone loss of the peri-implant bone crest for the PA group (control) between implant placement and 6 months was 0.23 ± 0.29 mm. After 12 months, the result was 0.21 ± 0.27 mm. The radiographic measurement of the mean bone loss of the peri-implant bone crest for the DA group (study) between implant placement and 6 months was 0.14 ± 0.18 mm. After 12 months, the result was 0.14 ± 0.21 mm. For the control group, there are statistically significant differences over time (*p* < 0.001) in total bone loss and comparing the different moments it appears that there are differences statistically significant between day zero and 6 months (*p* = 0.002) and between day zero and 6 12 months (*p* = 0.012), but not between 6 months and 12 months (*p* = 1,000). For the study group, there are statistically significant differences over time (*p* < 0.001) in total bone loss and comparing the different moments it appears that there are differences statistically significant between day zero and 6 months (*p* = 0.010) and between day zero and 6 and 12 months (*p* = 0.034), but not between 6 months and 12 months (*p* = 1,000). There are no statistically significant differences (*p* = 0.559) in total bone loss on day zero between the control and study group. There are no statistically significant differences (*p* = 0.442) in total bone loss after 6 months between the control and study groups. There are no statistically significant differences (*p* = 0.330) in total bone loss after 12 months between the control and study group—see Table 6.

## 4. Discussion

The present study, considering an implant with conical connection and platform switching installed in healed alveolar ridges, submitted to a delayed loading protocol and rehabilitated with screw-retained crowns, demonstrated that the placement of the definitive abutment immediately after the implant surgery and no longer removing it was equivalent to the conventional protocol in which the abutment was disconnected and reconnected at least three times during the period comprehended between implant placement and connection of the definitive abutment (*p* = 0.442; group DA: 0.14 mm vs. group PA: 0.23 mm), as well as between 6 and 12 months (*p* = 0.330; group DA: 0.14 mm vs. group PA: 0.21 mm). The null hypothesis of the present investigation considered that no difference would be observed in bone loss (or gain) between the 2 groups after implant placement. After 12 months, there was a non-significant difference concerning the marginal bone level between the two groups. Therefore, the null hypothesis of the study was accepted as valid.

The results of our trial confirm findings reported in other clinical studies with similar follow up periods or longer than 12 months [38,46,47,48].

In a recent clinical study published by Praça [48], the authors concluded that the installation of the definitive abutment at the time of implant placement did not prevent peri-implant marginal bone loss after 12 and 24 months when compared with the conventional protocol in which the healing abutment was disconnected and reconnected 3 times between the surgical procedure and the connection of the final abutment. After 24 months, the bone loss was 0.61 ± 0.1 mm for the DA group and 0.81 ± 0.15 mm for the PA group, with no statistically significant difference between the two groups.

In another study, Koutouzis and collaborators [46] also did not observe any significant difference in peri-implant marginal bone loss between the test group and the control group at 3 months (0.07 mm vs. 0.12 mm) and at 6 months (0.13 mm vs. 0.28 mm), corroborating the results of Degidi [38]. In the control group, Degidi and coworkers [38], removed and reconnected the abutments for 4 times during the period between implant surgery and the connection of the final prosthesis. After 36 months, no statistically significant difference was reported between the groups regarding changes in the vertical bone level.

A major difference between the two aforementioned studies is the surgical protocol for implant placement. Koutouzis [46] only included implants placed in healed ridges just like the studies conducted by Grandi [39], Molina [49], and Praça [48], with the implant–abutment connection (IAC) positioned epicrestally. Degidi and collaborators [38] followed a post-extraction implant placement protocol, positioning the implant platform 1 mm beneath the crestal bone level. The same approach was also followed by Canullo et al. [31], Grandi et al. [37], and Luongo et al. [50]. The subcrestal positioning of the implant may act as a confounder in assessing the possible influence of the abutment connection. In our study, all implant platforms should be positioned epicrestally [51,52]. However, alveolar ridges are rarely flat, which makes it difficult to position them at the same level around the entire perimeter of the implant platform. The flattening of the bone crest was allowed in a way to achieve a more homogeneous platform positioning. However, this procedure was not carried out in any region in a way to avoid other bone aggression factors that could stimulate resorption [53,54,55]. It was decided to place the implants subcrestally whenever necessary. Despite that, a homogenous population was obtained with no differences between the groups regarding the implant platform distance to the bone crest.

Other clinical trials designed to assess the one-abutment one-time concept included implants installed in post-extraction sockets [31,36,37], with two of them reporting less marginal bone loss for implants in which the abutment was placed and never removed [31,37]. Canullo and coworkers [31] randomized immediate implants to receive definitive abutments versus provisional abutments that have been removed at least 2 times before connecting the final abutment. Thirty-six months after the delivery of the final prosthesis, they reported statistically significant greater bone loss in the control group (control group: 0.55 mm; test group: 0.34 mm). Grandi [37] reported peri-implant marginal bone resorption of 0.065 mm after 6 months and 0.094 mm at 1 year after surgery for the test group and 0.36 mm (6 months) and 0.44 mm (1 year) for the control group. In other studies that included post-extraction sockets and healed ridges [47,50,56], no differences were observed in the 4 months follow-up appointment [50] but increased bone loss was reported after 1 and 3 years for the groups submitted to repeated abutment disconncetions and reconnections [47,56]. The one-abutment one-time concept is a valid option compatible with the implant placement in post-extraction sites protocol [31] in which the connection of a definitive abutment can favor the sealing of the alveolus by means of the immediate installation of the provisional rehabilitation. On the other hand, alongside tooth extraction, the initial bone remodeling process takes place mediated by multiple cofactors such as the thickness of the buccal bone wall [57]. The post-extraction implant placement protocol may not be a good model to assess the impact of the one-abutment one-time concept, since there are significant changes resulting from the resorption of the crest, both vertical and horizontally, during alveolar healing, which are not prevented by immediate installation of the implant [58,59]. The 3D positioning of the implant in the post-extraction socket also significantly influences the alveolar bone remodeling [60]. It is also prudent to mention that the majority changes in soft and hard tissues occur during the first three months after implant surgery [61], with the changes associated with immediate implant placement protocol being usually more unpredictable. Therefore, the decision to include in our study only implants placed in healed ridges seems to have been the most appropriate to assess the effect of abutment disconnection and reconnection on the peri-implant marginal bone level.

With respect to the loading protocol, there is also heterogeneity in the published literature on the one-abutment one-time concept. In a systematic review, Esposito [62] compared immediate with conventional loading. The authors reported an average marginal bone loss of 0.1 mm more in implants submitted to conventional loading when compared with implants submitted to immediate loading. Moreover in 2013, Nicolau and coworkers [63] published the results of a prospective, randomized, multicenter study to assess radiographic changes at the bone crest level between the day of surgery and 3 years in implants submitted to immediate or early loading (28 to 34 days after surgery). The variation was 0.9 mm for the immediate loading group and 0.57 mm for the early loading group. However, the more intense remodeling occurred during the first 5 months (connection of the final restoration), with the immediate load group losing, on average, 0.82 mm and the early load group only 0.56 mm. Contrary to what was observed in the study of Grandi [39], which included implants submitted to immediate loading, our study followed a conventional loading protocol in way to avoid the introduction of confounding variables.

One of the inclusion criteria of our study was to allocate the implant to one of the groups only if it achieved primary stability. All implants reached primary stability, with an average ISQ value of 80.9 on the day of surgery. After the healing period, the mean value of the ISQ was 80. The average of the ISQ values obtained on the day of implant placement was 82.6 for the DA group and 79.6 for the PA group. On the day of impression taking, these values were 79.9 (DA) and 81.6 (PA). At 6 months, the ISQ values were 79.7 (DA) and 79.4 (PA) and at 12 months, 81.3 (DA) and 80.2 (PA). The abutment placement protocol did not influence the variation of the ISQ.

In our study, all implants were rehabilitated with screw-retained stock abutments with the purpose to avoid the inclusion of cement in the peri-implant biological space [64,65,66] and its potential complications [64,65,66,67,68,69], as well as to avoid great variations in the design of the sub-gingival contour inherent to the use of customized abutments. Indeed, some studies do not mention the type of retention of implant-supported crowns [31,36,50]. Others report the rehabilitation with cemented crowns [39,46].

Regarding other clinical parameters analyzed, such as the patient’s oral hygiene, the plaque index, the probing bleeding and probing depth, there were no statistically significant differences between the two groups.

During continuous disconnections and reconnections, microbiologically contaminated fluid products can easily reach the implant chamber, creating an environment conducive to anaerobic bacterial flora. In our study, whenever the healing abutment was disconnected, measures were followed to protect the implant from potential exposure to oral fluid. Furthermore, on the day of the surgery all the healing abutments connected were new and whenever they were removed (control group) they were always disinfected with chlorhexidine 0.12% before being reconnected. This aspect may have been very important in the good results shown by the control group, which were similar to the results obtained in the test group. Most of the published studies do not mention or specify how the abutment disinfection was performed and how the implant chamber was protected once exposed to the oral environment. Many trials also fail to report whether healing abutments are reused. Some evidence begins to emerge that discourages the reutilization of prosthetic components [70,71]. Bidra and coworkers [70] conclude that the methods routinely used for the cleaning and sterilization of reused titanium healing abutments are not fully effective in the complete removal of contaminating agents; however, until now there are no reports of any harmful consequences for the bone–implant interface or for the patient systemic condition.

Several systematic reviews recently published are unanimous about the need of standardization of comparable parameters between groups in future studies on the effect of repeated disconnection and reconnection of abutments in peri-implant marginal bone resorption [70,71,72,73,74,75,76,77]. Among such heterogeneity, the type of implant/implant–abutment connection, the macro design, and the location of the implant in the arch and the surgical placement protocol of the implant seems to have a greater influence on the initial bone remodeling [78]. Other etiological factors associated with peri-implant bone loss should also be contemplated in the design of the studies, namely, the stability of the connection, the abutment decontamination and asepsis, the associated load protocol [79], variations in operator experience, bone loss assessment methods, bone quality and initial thickness of gingival tissue, and the use of bone substitute materials, as they may influence the treatment outcome [73].

Likewise, it is also consensual that this standardization should also extend to the follow-up time. In some publications, the short follow-up periods may probably have affected the results, negatively influencing the quality of the reported evidence, considering that some studies present less than 6 months of follow-up. In our study, it was established a follow-up period of 12 months, supported by Buser’s success criteria that points to a maximum bone loss of 1.5 mm at the end of the first year after implant placement, stabilizing from that point on.

It is also desirable that such studies are designed in such a way that they do not only report parameters related to soft and hard tissue, but also aesthetic results and patient satisfaction, taking in consideration all confounding variables, in particular the frequency of the provisional abutment disconnections and reconnections, in a way to confirm or refute the current evidence [73]. Our study implemented rigorous inclusion and exclusion criteria that allowed some comparisons, removing probable confounding variables, based on a sample with considerable homogeneity procedures and assessed parameters, as well as techniques for evaluating these parameters. However, we recognize the limitations associated with the adoption of standardized protocols due to the variety of implant systems, rehabilitation techniques, and equipment available for measurement of the assessed parameters.

Our clinical trial achieved a 100% implant and prosthetic success rate at the 12-month follow-up examination for both the study and the control group.

Although the difference verified between the two groups is enough to compromise neither the success nor the survival of the implants, the results of this study may serve to help the clinician to better understand the biomechanical behavior and to have a more precise perspective on the effects on peri-implant tissues when deciding between the one-time one-abutment protocol and the conventional protocol of repeated abutment disconnections to rehabilitate an implant in a given clinical situation.

One limitation of the present study could be the sub-crestal placement of some implants in way to avoid flattening of the bone crest, making it impossible to reach a uniform implant depth placement and therefore making it difficult to be compared with other studies that clearly guarantee the same depth of implant placement around the implant platforms. In our study, single and multiple splinted crowns were included. Before analyzing the data, the potential differences related with these two variables were assessed reporting a good distribution between the two groups and no significant interaction with the main outcome was found. Although, we think whenever possible it would be preferable not to combine single and multiple units. Another aspect related with our study is the height of the abutment. The selection was not performed randomly, as determined by the judgement of the clinician. Although, regardless of the group, no statistically significant differences were observed for total bone loss between groups defined by abutment height, it would be desirable for future studies to use abutments of the same height whenever possible.

## 5. Conclusions

After a period of 12 months, the placement of the final abutment at the time of implant placement no longer removing it has demonstrated at least an equivalent effect on the maintenance of the marginal peri-implant bone level when compared with the conventional protocol that consists at least in three repeated disconnections and reconnections of the healing abutment.

## Figures and Tables

**Table 1:** Baseline demographic characteristics of the study sample.

	Treatment Group			
	PA (Control, *n* = 30)	DA (Test, *n* = 23)	Total (*n* = 53)	*p*-Value
Sex (*n* and % of subjects)				
Male	9 (30%)	9 (39.1%)	18(34%)	0.565
Female	21 (70%)	14 (60.1%)	35(66%)	
Center (*n* and % of subjects)				
07	15 (50%)	10 (43.5%)	25 (42.2%)	0.782
08	15 (50%)	13 (56.5%)	28 (52.8%)	
Age (years)				
Median	49.5	50.9	50.7	
Range	35–61	32–67	32–67	
Smoking (*n* and % of subjects)				
Non-smoker	26 (86.7%)	21 (91.3%)	47	0.687
Smoker < 10 cigs/day	4 (13.3%)	2 (8.7%)	6	
Diabetes				
No	28 (93.3%)	23 (100%)	51	0.499
Yes	2 (6.7%)	0 (0.0%)	2	
ASA classification				
Type I	25 (83.3%)	21 (91.3%)	46	
Type II	4 (13.3%)	0 (0.0%)	4	
Type III	1 (3.4%)	2 (8.7%)	3	
Number of implants placed				
Single units	15 (50.0%)	13 (56.5%)	28	0.782
Multiple units	15 (50.0%)	10 (43.5%)	25	
Implants position				
Maxilla	10 (33.3%)	11 (47.8%)	21	
Mandible	20 (66.7%)	12 (52.2%)	32	
Bone Quality				
Type I	0 (0.0%)	0 (0.0%)	0	1.000
Type II	10 (33.3%)	7 (30.4%)	17	
Type III	20 (66.7%)	16 (69.6%)	36	
Type IV	0 (0.0%)	0 (0.0%)	0	
Implant diameter				
4 mm	23	17	40	1.000
4.5 mm	7	6	13	
Implant length				
8	11	6	17	0.647
10	11	11	22	
12	8	6	14	
Primary stability (No/Yes)	0/30 (0.0%/100%)	0/23 (0.0%/100%)	0/53	
Primary rotational stability (No/Yes)	0/30 (0.0%/100%)	0/23 (0.0%/100%)	0/53	
Resorbed ridge classification				
Converging walls	15 (50.0%)	9 (39.1%)	24	0.170
Parallel walls	15 (50.0%)	11 (47.8%)	26	
Ridge with undercuts	0 (0.0%)	3 (13.0%)	3	
Knife-edged ridge form	0 (0.0%)	0 (0.0%)	0	
Implant platform position (*n* and % of subjects)				
Epicrestal	12 (40.0%)	7 (30.4%)		0.569
Sub-crestal	18 (60.0%)	16 (69.4%)		

**Table 2:** ISQ values at the day of the surgery, 8 weeks, 6 and 12 months for both groups.

		DA	PA	TOTAL	*p*
ISQ-1	N	23	30	53	
	Minimum	70	63	63	
	Maximum	91	88	91	
	Average	82,6	79,6	80,9	0.570
	SD				
ISQ-2	N	23	30	53	
	Minimum	57	74	57	
	Maximum	93	90	93	
	Average	77,9	81,6	80	0.102
	SD				
ISQ-3	N	23	30	53	
	Minimum	69	71	69	
	Maximum	97	97	97	
	Average	79,7	79,4	80,8	0.360
	SD				
ISQ-4	N	23	30	53	
	Minimum	71	72	71	
	Maximum	98	95	98	
	Average	81,3	80,2	80,7	0.628
	SD				

**Table 3:** Modified Plaque Index, 6 and 12 months.

mPLI	Group				Group			
	DA 6 Months	PA 6 Months	Total	*p*	DA 12 Months	PA 12 Months	Total	*p*
Visible with probe	21	22	43	0.225	19	24	43	0.767
Visible	2	6	8		1	3	4	
Abundant	0	2	2		3	3	6	
Total	23	30	53		23	30	53	

**Table 4:** Modified sulcus bleeding index, 6 and 12 months.

Modified Bleeding Index	Group				Group			
	DA 6 Months	PA 6 Months	Total	*p*	DA 12 Months	PA 12 Months	Total	*p*
Punctual	22	26	48	0.374	23	29	52	1.000
Line in groove	1	4	5		0	1	1	
Total	23	30	53		23	30	53	

**Table 5:** Probing Depth, 6 and 12 months.

PD	Group			*p*	Group			*p*
	DA 6 Months	PA 6 Months	Total		DA 12 Months	PA 12 Months	Total	
0	18	29	47	0.074	19	27	36	0.451
1	5	1	6		4	3	7	
Total	23	30	53		23	30	53	

**Table 6:** Total bone level changes/groups.

		0 Months	6 Months	12 Months
Group PA (control)	mean ± SD	0.05 ± 0.08	0.23 ± 0.29	0.21 ± 0.27
	median (p25;p75)	0.00 (0.00, 0.08)	0.14 (0.00, 0.32)	0.13 (0.00, 0.26)
Group DA (test)	mean ± SD	0.03 ± 0.06	0.14 ± 0.18	0.14 ± 0.21
	median (p25;p75)	0.00 (0.00, 0.03)	0.05 (0.00, 0.27)	0.03 (0.00, 0.24)

## Data Availability

Data sharing is not applicable to this article.

## Abbreviations

ISQ	implant stability quotient
DA	definitive abutment
PA	provisional abutment
MBL	marginal bone loss
PS	platform switching
FDI	world dental federation
TMJ	temporomandibular joint
JPEG	joint photographic experts group
DIB	distance between the implant platform and the first visible bone contact
ICC	intraclass correlation coefficient
TOST	two one-sided tests
mPLI	modified plaque index
